# Tolerance of uncertainty and reflection ability among medical students in Germany

**DOI:** 10.1186/s12909-026-10455-9

**Published:** 2026-09-23

**Authors:** Christopher Holzmann-Littig, Nana Jedlicska, Pascal O. Berberat, Marjo Wijnen-Meijer

**Affiliations:** 1 Nephrocare Friedberg GmbH, Friedberg, Germany; 2 Department of Psychosomatic Medicine and Psychotherapy, Technical University of Munich, Langerstr. 3, Munich, 81675 Germany; 3 Medical Education Center, School of Medicine and Health, Technical University of Munich, Munich, Germany; 4 Institute of Medical Education, Faculty of Medicine Carl Gustav Carus Dresden, Dresden University of Technology, Dresden, Germany

**Keywords:** Uncertainty tolerance, Reflection ability, Medical students, Medical education

## Abstract

**Background:**

With growing recognition of uncertainty tolerance (UT) and reflection ability (RA) as core competencies for healthcare professionals, targeted promotion of these skills has become increasingly important in medical education. However, research on the associations between UT and RA and students’ individual characteristics, such as age and gender, remains limited, and existing findings are inconsistent. Addressing this gap, the present study assesses UT and RA among medical students at entry to medical school, examines their associations with age and gender, and explores implications for the tailored design of educational interventions.

**Methods:**

All first-year medical students at Ludwig Maximilian University and the Technical University of Munich were invited to participate in the survey during a compulsory joint course, *Introduction to Clinical Medicine*. The questionnaire, administered via EvaSys, included the Groningen Reflection Ability Scale, the Uncertainty Tolerance Scale, and demographic data.

**Results:**

A total of 761 participants were included in the analysis. The age could be determined for 384 participants, and the gender for 493 participants. Measuring UT resulted in a mixed picture: while students reported openness toward novel experiences, they simultaneously expressed a preference for predictability and routine. Linear regression analysis revealed no significant association between overall UT scores and age or gender (all *p* > 0.05). However, statistically significant gender differences emerged at the item level, with female students reporting lower tolerance of uncertainty. Regarding RA, linear regression analysis similarly revealed no significant association between overall GRAS scores and age or gender (R² = 0.002; all *p* > 0.05). However, statistically significant gender differences were identified at the item level, with female students reporting a higher level of self-underestimation and self-doubt.

**Conclusions:**

Although overall UT and RA scores were not significantly associated with gender or age, exploratory item-level analysis suggested gender-specific differences in certain aspects of both constructs. Given the high proportion of female students, these differences may warrant further investigation and consideration in curriculum development to promote equitable opportunities and optimally prepare all students for clinical practice.

**Supplementary Information:**

The online version contains supplementary material available at https://doi.org/10.1186/s12909-026-10455-9.

## Background

Uncertainty is inherent to medical practice, arising from ambiguous clinical findings, complex decision-making, and unpredictable treatment outcomes [1–3]. Uncertainty tolerance (UT) refers to how individuals interpret and respond cognitively, emotionally, and behaviourally to situations characterized by ambiguity, probability, and complexity. It is therefore regarded as a core competency for healthcare professionals. How someone experiences uncertainty depends on several moderators, including characteristics of the uncertainty stimulus (e.g., type and source), individual characteristics (e.g., personality traits, abilities, skills, and motivations), situational factors (e.g., features of clinical encounters such as time pressure and patient–clinician communication), cultural factors (e.g., values, norms, and beliefs), and social context factors (e.g., community and institutional resources, including interpersonal relationships and the availability of institutional support) [4]. Evidence indicates that lower UT is associated with impaired psychological well-being, including a higher risk of burnout and stress among medical students and physicians [5–8]. Moreover, low UT is linked to inappropriate use of healthcare resources and medical errors among physicians [1, 4, 7, 9]. Geller et al. reported that UT does not change during medical school and argued that it should be considered a prerequisite for admission [10]. Other studies suggest that UT may be trained, and that undergraduate medical education may provide an ideal context for enhancing it [11–13]. Accordingly, UT has become a prominent focus in medical education and research in recent years [14]. A range of educational interventions has been developed to enhance medical students’ UT, including problem-based learning curricula, medical humanities courses, and simulation-based training [2]. Much of the research has focused on conceptualizing and measuring UT, as well as on exploring how psychological factors and individual predictors relate to it [8, 15]. Qualitative studies further investigate how students experience uncertainty in clinical contexts and how educational environments might shape UT development over time [13, 16, 17]. Together, these findings suggest that students’ individual characteristics, including gender, age, educational background, discipline, and stage of training, influence how learners engage with and manage uncertainty. However, findings remain heterogeneous. While some studies report higher UT among older and female medical students [18], others report higher UT among younger and male students [10, 19]. Further studies show no differences regarding gender [20, 21].

Furthermore, reflection skills are increasingly recognized as essential competencies for healthcare professionals working in complex, evolving healthcare systems [22]. Reflection is a metacognitive process in which individuals revisit and critically examine their experiences, thoughts, and actions in order to interpret and learn from them. This sense-making process, usually triggered by a specific event or situation, leads to greater understanding and awareness that can inform future action [22, 23]. Reflection serves multiple purposes. It is an essential component of self-regulated and lifelong learning, and it is also required for the development of therapeutic relationships and professional expertise [23]. Fostering reflective capacity in medical education enhances critical thinking, clinical reasoning, and professionalism among medical students and trainees [24, 25]. Studies indicate that reflective ability is associated with improved communication skills [26] and with greater empathy or preservation of empathy among medical students [27]. Reflective practice may also foster collegiality and reduce feelings of isolation among students, although direct evidence of its effects on well-being remains limited [27]. Accordingly, reflective practice is increasingly embedded in medical curricula [22, 23]. Various educational approaches are employed to promote reflection, including text-based reflective journals, critical incident reports, creative photo-elicitation techniques, online reflective groups, and storytelling [24, 27]. A few studies have investigated the relationship between reflective ability and individual characteristics of medical students. A cross-sectional study of Iranian medical students found no statistically significant association between age and reflective ability and reported no significant gender differences [28]. In line with these findings, studies in postgraduate medical education also suggest that reflective ability is not systematically influenced by gender or year of training [29]. Overall, the number of studies examining the relationship between tolerance of uncertainty or reflective ability and individual character traits of medical students, such as age and gender, remains limited. Moreover, the available findings are contradictory and do not allow for clear conclusions. Aiming to close the research gap and provide a solid foundation for the differentiated consideration of personal characteristics in the design of learning formats, this article addresses the following research questions: (1) What level of uncertainty tolerance and reflection ability do medical students exhibit at the beginning of their studies? (2) How are age and gender associated with medical students’ tolerance of uncertainty and reflective capacity? (3) How can possible differences related to individual characteristics inform course design?

## Methods

The data were collected on 14 October 2019 during a class that took place that day as part of a larger cross-sectional study [30]. All new medical students at Ludwig Maximilian University and Technical University of Munich are required to complete a joint compulsory course, *Introduction to Clinical Medicine*. A total of 918 students were enrolled in the course in 2019, and all were invited to participate in this survey study. Students were informed that participation in this study was voluntary and that successful course completion did not depend on participation. The survey was paper-based and prepared using EvaSys (EvaSys Central Evaluation 8.0). The survey included the Groningen Reflection Ability Scale (GRAS), a 23-item instrument to measure medical students’ self-reported personal reflection ability. The GRAS is a one-dimensional scale covering three relevant aspects of personal reflection: self-reflection, empathetic reflection, and reflective communication. Items are rated on a 5-point Likert scale (1 = totally disagree to 5 = totally agree), with five items (3, 4, 12, 17, and 21) reverse-scored. Total scores range from 23 to 115, with higher scores indicating greater reflective capacity [31]. This study used a German-language translation of the GRAS. The original Dutch questionnaire was translated into German and cross-checked against an independent German translation of the English version. The two German translations showed a high degree of agreement, and the resulting German version was administered to participants. For each participant, a mean GRAS Score was calculated as the average of all item responses after inverting the above-mentioned items. Uncertainty tolerance was measured using the German 8-item Uncertainty Tolerance Scale (UTS) developed by Dalbert (2002) [32]. The UTS assesses individuals’ ability to accept and cope with uncertain situations. Items are rated on a 6-point scale ranging from 1 (strongly disagree) to 6 (strongly agree). Three items (2, 5, and 8) are negatively worded and reverse-scored before summing, with higher scores indicating greater uncertainty tolerance. The validated German version of the UTS was administered in this study. In addition, the questionnaire included items on the year of birth and gender.

Statistical analysis was performed using JASP (JASP version 0.96). Participants aged 50 or older were excluded from the statistical analysis as they constituted a very small subgroup and represented extreme values within the sample’s age distribution, potentially exerting disproportionate influence on the results. Additionally, participants who answered fewer than four items across the UT and RA scales were excluded from all analyses. In several cases, school grades were not provided or illegible and therefore not included in the study. Normal distribution was tested using the Shapiro-Wilk test. To verify the consistency of the items, Cronbach’s alpha was calculated for both scales. An exploratory multiple linear regression analysis was conducted to investigate possible associations between the sociodemographic factors of age and gender and uncertainty tolerance and reflection ability. Only cases with provided information on age and gender were included in the respective analysis. The respective scores for the items were used as the dependent variable, and age and gender were included in the regression model as independent variables. Since no specific hypotheses regarding the influencing factors were formulated due to the exploratory nature of the study, the analysis served to identify possible associations and estimate their effect sizes. Due to the exploratory nature of the study, Bonferroni correction was not applied; this should be considered when interpreting the results.

This study was reported in accordance with the STROBE (Strengthening the Reporting of Observational Studies in Epidemiology) guidelines for cross-sectional studies [33].

## Results

### Sample characteristics

**Fig. 1:**
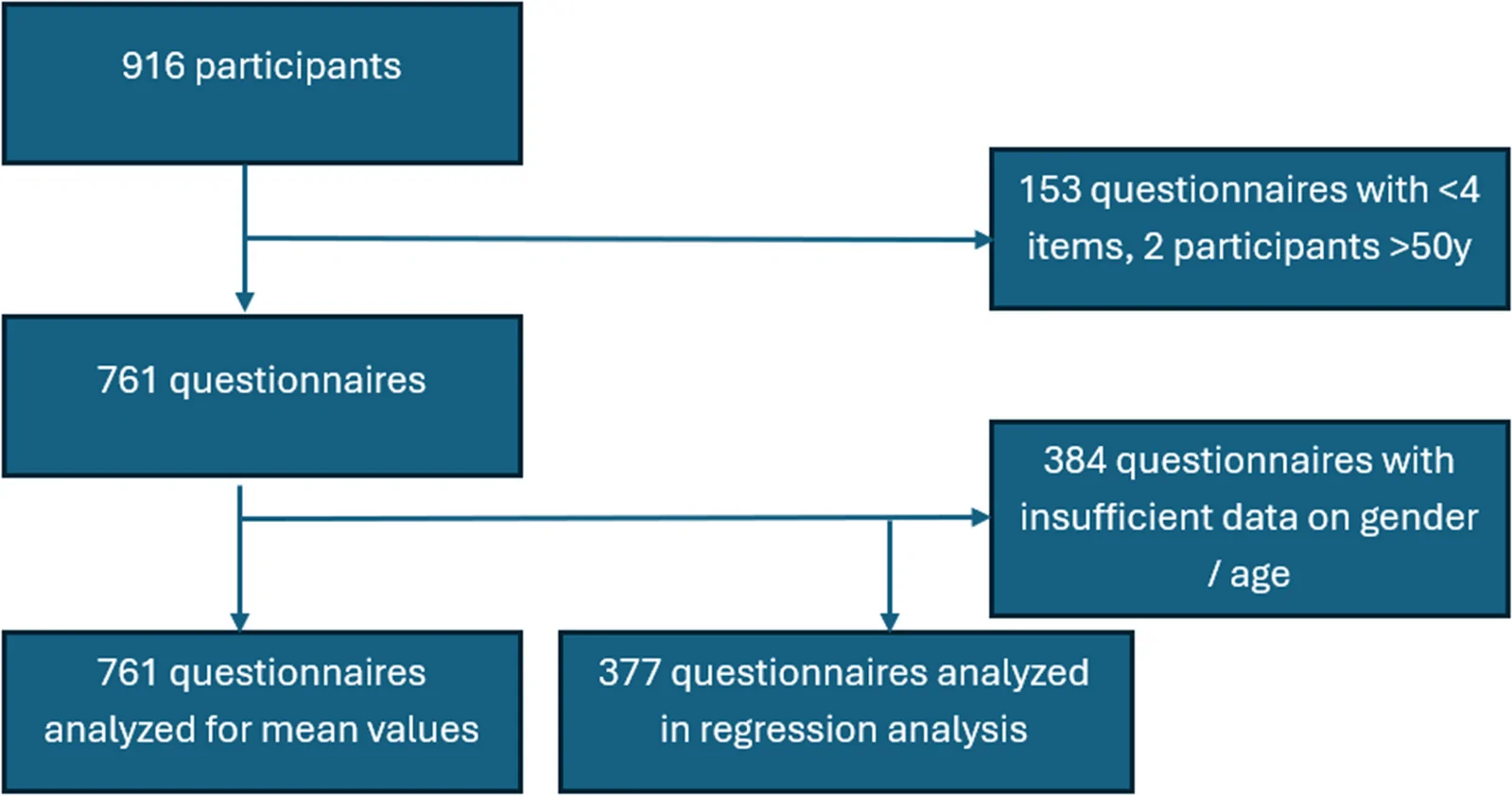
Flowchart of exclusion of participants

916 participants answered the survey. Of those, 153 participants had terminated the questionnaire, which also included other scales being surveyed [30] before answering at least four items of the UT and RA scales. Another two participants were more than 50 years old. Eventually, 761 participants were included in this analysis (see Fig. 1).

Due to missing information or poor legibility, the age could be determined for 384 participants, and the gender for 493 participants. Participants’ median age was 19 years, IQR 3, with a minimum age of 16 and a maximum of 34 years. 329/493 (66.7%) participants were female. This reflects the expected age and gender distribution for this semester. No statistically significant differences regarding age (*p* = 0.378) were observed between female and male students.

### Uncertainty tolerance

Measuring the uncertainty tolerance resulted in a mixed picture. The item “I like to try things out, even if nothing comes of it” received clear approval with a mean score of 4.8 points. Conversely, students also agreed with the item “I like to know what to expect” with a mean score of 4.6 points. Students also seem to prefer orderly structures; the item “When things around me are all topsy-turvy, I am quite all right” received low agreement, with a mean of 3.1 points. The solubility of tasks, on the other hand, seemed less important to students; the item “I like to engage in tasks for which there is a solution” received practically no agreement, with a mean of 2.3 points (see Table 1). In line with this, Cronbach’s alpha also showed only moderate consistency of the scale with 0.70. A total of 675 students (88.7%) provided responses to all eight uncertainty tolerance items. The number of respondents for each item is presented in Table 1.

**Table 1:** Students’ uncertainty tolerance, mean values. 761 questionnaires analyzed, 675 questionnaires with complete answers on all items. The *N* for each item is presented in the table

Item	Mean	SD	Min	Max
I like to try things out, even if nothing comes out of it. (*N*** = **745)	4.846	1.096	1.000	6.000
I like to engage in tasks for which there is a solution. (*N*** = **749)	2.252	1.105	1.000	6.000
I like unexpected surprises. (*N*** = **751)	4.009	1.399	1.000	6.000
I like to let things happen. (*N*** = **747)	3.987	1.456	1.000	6.000
When I work or study, I like things to be predictable and routine. (*N*** = **739)	3.708	1.424	1.000	6.000
I like change and excitement. (*N*** = **733)	4.080	1.270	1.000	6.000
When things around me are all topsy-turvy, I am quite all right. (*N*** = **742)	3.053	1.488	1.000	6.000
I like to know what to expect. (*N*** = **746)	4.552	1.134	1.000	6.000

 A linear regression was calculated to examine the relationship between gender and age and the items of uncertainty tolerance. For the UTS total score, no significant differences regarding age and gender were observed. However, the model was significant for the item “I like change and excitement”, but explained only a small proportion of the variance, with older students agreeing less strongly. Also, for the item “I like to know what to expect,” the model was significant but explained only a small proportion of the variance; gender was also a significant predictor: female students agreed with this item more strongly. For the item “I like to engage in tasks for which there is a solution, the model showed no significant association overall; however, female students agreed with this statement significantly more often. The same was the case for the item “When things around me are all topsy-turvy, I am quite all right.” The model showed no significant overall association; however, a statistically significant relationship was observed for this item, with female students disagreeing significantly more often than their male counterparts (see Table 2).

**Table 2:** Relationship between age/gender and uncertainty tolerance, results of linear regression analysis. B: unstandardized coefficient, ß: standardized coefficient, R²: coefficient of determination, indicating the proportion of variance in the dependent variable explained by the regression model, F(df): F-statistic with degrees of freedom. 377 questionnaires were included, the respective number of analyzed questionnaires per item is given as *N*

Predictor	B	SE	ß	t	*P*
Uncertainty Tolerance Scale Total Score (*N* = 335) R² 0.007, F (df) 1.222 (2, 332), p (model) 0.296					
Gender (female)	− 1.089	0.702		-1.553	0.121
Age	0.011	0.099	0.006	0.108	0.914
I like to try things out, even if nothing comes out of it. (*N*** =** 364) R² 0.007, F (df) 0.7362 (2, 361), p (model) 0.563					
Gender (female)	-0.072	0.119		-0.603	0.547
Age	0.015	0.017	0.045	0.855	0.393
I like to engage in tasks for which there is a solution. (*N*** = **365) R² 0.011, F(df) 1.990 (2, 362), p (model) 0.138					
Gender (female)	0.240	0.121		1.988	0.048
Age	-0.001	0.017	-0.003	-0.066	0.947
I like unexpected surprises. (*N*** = **370) R² 0.00, F(df) 1.304 (2, 367), p (model 0.273)					
Gender (female)	0.213	0.154		1.383	0.167
Age	-0.017	0.022	-0.039	-0.755	0.451
I like to let things happen. (*N*** = **366) R² 0.001, F(df) 0.251 (2, 363), p (model) 0.778					
Gender (female)	-0.047	0.163		-0.290	0.772
Age	0.015	0.023	0.033	0.628	0.530
When I work or study, I like things to be predictable and routine. (*N*** = **364), R² 0.010, F(df) 1.851 (2, 361), p (model) 0.159					
Gender (female)	0.267	0.158		1.684	0.093
Age	-0.018	0.022	-0.043	-0.822	0.412
I like change and excitement. (*N*** = **361) R² 0.029, F(df) 5.259 (2, 358), p (model) 0.006					
Gender (female)	0.013	0.142		0.091	0.928
Age	-0.065	0.020	-0.169	-3.231	0.001
When things around me are all topsy-turvy, I am quite all right. (*N*** = **361), R² 0.028, F(df) 5.231 (2, 358), p (model) 0.006					
Gender (female)	-0.526	0.163		-3.229	0.001
Age	-0.008	0.023	-0.019	-0.365	0.715
I like to know what to expect. (*N*** = **364), R² 0.022, F(df) 4.021 (2,361), p (model 0.019)					
Gender (female)	0.334	0.125		2.676	0.008
Age	-0.014	0.018	-0.042	-0.812	0.417

### Reflection ability

Results on the Groningen Reflection Ability Scale were more consistent. All items positively correlating with reflection ability received high ratings, with a mean of 3.8–4.6 points on a five-point Likert scale. Only, the item “sometimes others say that I overestimate myself” that (based on the original publication) [31] should not be inversely coded received a mean of 1.9 points. The inversed items “I reject different ways of thinking“, „I do not like to have my standpoints discussed”, “I sometimes find myself having difficulty in illustrating an ethical standpoint”, “I sometimes find myself having difficulty in thinking of alternative solutions“ and “I do not welcome remarks about my personal functioning“ were disagreed by students with 1.4–2.8 points on the five-point Likert scale (see Table 3).

**Table 3:** Students’ reflection ability, mean values, SD: standard deviation

Item	Mean	SD	Min	Max
I want to know why I do what I do. (*N*** =** 723)	4.567	0.646	2.000	5.000
I am aware of the emotions that influence my behavior. (*N*** = **725)	4.154	0.733	1.000	5.000
I do not like to have my standpoints discussed. (*N*** = **716)	2.292	1.139	1.000	5.000
I do not welcome remarks about my personal functioning. (*N*** =** 720)	2.310	0.945	1.000	5.000
I take a closer look at my own habits of thinking. (*N*** = **715)	4.372	0.699	2.000	5.000
I am able to view my own behavior from a distance. (*N*** = **708)	3.828	0.853	1.000	5.000
I test my own judgments against those of others. (*N*** = **704)	3.403	0.966	1.000	5.000
Sometimes others say that I do overestimate myself. (*N*** = **697)	1.891	0.987	1.000	5.000
I find it important to know what certain rules and guidelines are based on. (*N*** = **705)	4.237	0.801	1.000	5.000
I am able to understand people with a different cultural / religious background. (*N*** = **696)	4.315	0.763	1.000	5.000
I am accountable for what I say. (*N*** = **703)	4.526	0.628	1.000	5.000
I reject different ways of thinking. (*N*** = **699)	1.386	0.679	1.000	5.000
I can see an experience from different standpoints. (*N*** = **696)	4.208	0.682	2.000	5.000
I take responsibility for what I say. (*N*** = **696)	4.259	0.697	1.000	5.000
I am open to discussion about my opinions. (*N*** = **653)	4.386	0.628	2.000	5.000
I know my limitations. (*N* ** =** 675)	3.833	0.867	1.000	5.000
I sometimes find myself having difficulty in illustrating an ethical standpoint. (*N* ** = **661)	2.841	1.104	1.000	5.000
I am aware of the cultural influences on my opinions. (*N*** = **676)	4.030	0.812	2.000	5.000
I want to understand myself. (*N*** =** 668)	4.488	0.727	1.000	5.000
I am aware of the possible emotional impact of information on others. (*N*** = **670)	4.500	0.662	1.000	5.000
I sometimes find myself having difficulty in thinking of alternative solutions. (*N*** = **657)	2.572	0.957	1.000	5.000
I can empathize with someone else’s situation. (*N*** = **657)	4.408	0.704	1.000	5.000
I am aware of the emotions that influence my thinking. (*N*** = **658)	4.150	0.744	2.000	5.000

Linear regression analysis revealed no statistically significant association between the mean GRAS total score and students’ age or gender, F (2, 360) = 0.386, *p* = 0.680, R² = 0.002; the individual predictors did not prove to be significant (age: *p* = 0.599; gender: *p* = 0.499). However, in the regression analysis for each item, statistically significant gender differences were observed.

Female and older students showed less willingness to discuss their points of view. For the item “I do not like to have my standpoints discussed,” the regression model was significant, F (2, 350) = 6.140, *p* = 0.002, but it explained only a small proportion of the variance (R² = 0.034). Gender was a significant predictor (*p* = 0.006), as was age (*p* = 0.024).

In addition, the model was significant for the item “Sometimes others say that I do overestimate myself” (F (2, 347) = 13.88, *p* < 0.001, R² = 0.074) – here, only gender was significant (*p* < 0.001), with female students agreeing less with the item.

Female students also agreed significantly less with the item “I reject different ways of thinking” (*p* = 0.003), and here too there were no significant effects for age. Female students also reported more frequently that they wanted to understand themselves (*p* = 0.027) and that they were aware of the possible emotional impact of information on a person (*p* = 0.026).

## Discussion

### Uncertainty tolerance

Our study on first-year medical students’ tolerance for uncertainty yielded mixed results. First, students seem curious to try new things and do not necessarily require a clear solution to a task. On the other hand, students preferred things to be predictable and routine and disagreed with the item “When things around me are all topsy-turvy, I am quite all right.” Even more, they strongly agreed with the item “I like to know what to expect”. In both last-mentioned items, uncertainty tolerance was significantly lower among female students. These findings are consistent with previous literature. Nevalainen et al. reported that uncertainty was poorly tolerated by 11% of male students compared to 27% of female students [19]. However, the existing literature also includes findings that do not indicate a clear correlation between gender and UT [10]. Notably, in our study, a significant gender difference was observed for the item “When things around me are all topsy-turvy, I am quite all right,” which reflects emergency situations, with female students showing stronger disagreement with this statement. Also, the item “I like to know what to expect,” which reflects the opposite, was agreed with significantly more strongly by female students. Therefore, uncertainty tolerance training might be especially beneficial in early emergency training during medical studies, while considering gender differences. A current study showed that gender differences in learning and study strategies among medical students affect performance in both the preclinical curriculum and the USMLE Step 1 examination [34].

Although prior research has identified age-related differences in UT [10, 18], our findings did not support this association, except for the item “I like change and excitement.” One might expect differences in UT, as older students often have prior work experience and greater exposure to emergency situations. One possible explanation is that only first-semester students were invited to this study; however, age differences remained, as some participants had experienced a waiting period before admission to medical school. However, older students should not automatically be expected to have higher UT and, therefore, should not receive less training than their younger peers.

### Reflection ability

Regarding reflection ability, statistically significant differences were found across items for participants’ gender. Female students appeared to show slightly greater self-doubt, consistent with previous literature. Sethi et al. found that despite higher performance, female students rate their abilities similarly to male medical students, suggesting a tendency toward self-underestimation [35]. In our study, female students were less willing to discuss their points of view, which, at first glance, could be attributed to lower self-reflection. However, given the relatively high Cronbach’s alpha and the lack of significant differences in the overall average of the GRAS values, an alternative interpretation could be that female students preferred not to discuss their views due to uncertainty. This is underlined by the fact that female students significantly more often disagreed with the statement “I reject different ways of thinking” – suggesting that they had no problem with other opinions – but simply did not want to discuss their own opinions. Female students also agreed with the statement “I want to understand myself” significantly more often. Whether this should be interpreted as a sign of greater self-doubt or greater self-reflection can certainly be debated. However, given the literature, our results might indicate a higher level of self-underestimation or self-doubt among female students – these aspects could be reflected in future curricula [36].

The extent to which this potentially greater self-doubt among female students may be advantageous or disadvantageous remains a subject of debate. For example, female students reported being told by others that they would overestimate themselves significantly less frequently, which aligns with the findings of Stanek et al., who showed that confidence scores among plastic surgery trainees correlated more strongly with objective performance in women than in men [37]. Nevertheless, it seems advisable to take into account potential higher self-doubt among female students, as well as possible higher self-overestimation among male students, especially in emergency training contexts. At the same time, these differences should be continuously assessed and addressed throughout the entire course of study.

Taken together, these item-level findings should be interpreted with caution, given the lack of adjustment for multiple testing, and future studies are needed to confirm whether they reflect robust and generalizable gender differences.

### Strengths and limitations

One of the strengths of this study is the use of methodologically well-established scales. However, self-assessments capture perceived rather than actual competence and may not accurately reflect individuals’ actual competence levels. Studies show that students can both over- and underestimate their abilities, with self-evaluation accuracy varying by context and level of expertise. Therefore, the present findings should be understood as representing students’ perceptions of their competence rather than objective measures of their abilities. The early semester in which the study was conducted also appears advantageous for drawing conclusions about potential course offerings in the first years of study. Another positive aspect is that the age and gender structure of the sample is comparable to that of other German universities, which facilitates contextualizing the findings alongside comparable studies. However, since both variables tend to show little variation within this population anyway, their informative value regarding generalizability is limited.

However, the study also has weaknesses. First, multiple linear regression analysis was conducted in an exploratory manner, and the results should not be interpreted as causal relationships. To better reflect the exploratory nature of the study, also an independent samples t-test for the variable “gender” and a Pearson correlation analysis for “age” would have been an option. However, various different analyses would make the manuscript very difficult to read – we therefore decided to conduct an exploratory multiple linear regression. Furthermore, the Uncertainty Tolerance Scale was originally developed and validated as a composite measure, and we therefore analyzed the overall scale score – neither age nor gender were significantly associated with the total score. However, analyses at the item level revealed significant associations for selected items. Although these item-level findings should be interpreted with caution, they provide valuable insights into specific aspects of uncertainty tolerance. The item-level analyses, therefore, complement, rather than replace, the validated total-score analysis.

A limitation of this study is the amount of missing demographic data, which reduced the sample size available for analyses and may have affected the generalizability of the findings. Future studies may improve questionnaire design by using age categories rather than open-ended responses and providing more inclusive gender response options. Additionally, implementing measures that reduce the likelihood of skipped items without increasing participant burden may help. For instance, electronic questionnaires could include prompts for unanswered items, although such features should be used cautiously, as forced responses may increase the risk of inaccurate or random answers. Missing data were handled differently depending on the analysis. Participants with missing values on the sociodemographic variables age or gender were excluded from the regression analyses via listwise deletion, as these variables served as predictors in the models [38]. These participants were nonetheless retained for all other analyses where possible. Participants with isolated missing responses on questionnaire scales were also retained to minimize data loss, as excluding these cases would have resulted in the loss of data from 86 participants, most of whom had omitted only a single item. Moreover, no data were collected on potential occupations/student jobs or on prior vocational training, which could have influenced the results. In addition, the data were collected immediately prior to the onset of the COVID-19 pandemic; therefore, future research should examine whether self-uncertainty, reflective capacity, or tolerance of uncertainty have changed in the post-pandemic context. Nevertheless, our study may serve as a comparative baseline reflecting pre-pandemic conditions. Also, the students were not followed up for the rest of their studies, so possible changes in the impact of students’ age or growing medical knowledge on their uncertainty tolerance over time cannot be evaluated. Furthermore, follow-up studies should also consider the effects of potential teaching approaches that address the characteristics of students mentioned above.

The present study deliberately focused on students in the early stages of medical education, offering insight into UT and RA prior to substantial clinical exposure. Consequently, participants’ responses likely reflect prior educational experiences, personal expectations, and theoretically informed conceptions, rather than experience grounded in actual clinical practice. Furthermore, given their limited practical exposure, participants’ views may be more idealized than those of senior medical students or practicing physicians — an assumption consistent with prior research documenting a progressive decline in idealism as students advance through medical school [39]. We agree that a longitudinal follow-up would be a valuable direction for future research. Comparing students’ perceptions before and after substantial clinical exposure could shed light on how clinical practice shapes attitudes toward UT and RA, and whether the perspectives observed in the present study persist or evolve over time.

While age and gender were considered in the present study, other potentially relevant sociodemographic characteristics, such as ethnicity and socioeconomic background, were not assessed. These factors may also shape individuals’ attitudes toward UT and RA and could help explain heterogeneity in individuals’ responses. Future research should therefore consider a broader range of sociodemographic variables to achieve a more comprehensive understanding of the factors associated with UT and RA.

## Conclusions

In summary, the findings suggest that gender may be associated with certain aspects of medical students’ tolerance of uncertainty and self-reflection, with these differences emerging primarily at the item level rather than in overall UTS and GRAS scale scores. Given the high proportion of female students in medical education, further research is warranted to explore the educational relevance of these findings and whether they should be considered in the design of medical curricula that promote equitable educational and career opportunities and adequately prepare all students for clinical practice.

## Supplementary Information

Supplementary Material 1.

Supplementary Material 2.

## Data Availability

The data that support the findings of this study are available from the corresponding author (NJ) upon reasonable request.

## Abbreviations

UT: Uncertainty Tolerance
RA: Reflection Ability
GRAS: Groningen Reflection Ability Scale
UTS: Uncertainty Tolerance Scale

## References

[1] Babenko O, Lee A. Ambiguity and uncertainty tolerance and psychological needs of medical students: A cross-sectional survey. Clin Teach. 2022;19(6): e13523 10.1111/tct13523 PMID: 36000148. 36000148

[2] Patel P, Hancock J, Rogers M, Pollard SR. Improving uncertainty tolerance in medical students: A scoping review. Med Educ. 2022;56(12):1163–73 10.1111/medu14873 PMID: 35797009. PMC9796811 35797009

[3] Ghosh AK. Dealing with medical uncertainty: a physician’s perspective. Minn Med. 2004;87(10):48–51. PMID: 15559102. 15559102

[4] Hillen MA, Gutheil CM, Strout TD, Smets EMA, Han PKJ. Tolerance of uncertainty: Conceptual analysis, integrative model, and implications for healthcare. Soc Sci Med. 2017;180:62–75 10.1016/j.socscimed.2017.03.024 PMID: 28324792. 28324792

[5] Hancock J, Mattick K. Context Matters: The Impact of Context on Uncertainty Tolerance Scales in Academic Medicine. Acad Med. 2023; 98(8): 870 10.1097/ACM0000000000005229 PMID: 37146234. 37146234

[6] Stephens GC, Karim MN, Sarkar M, Wilson AB, Lazarus MD. Reliability of Uncertainty Tolerance Scales Implemented Among Physicians and Medical Students: A Systematic Review and Meta-Analysis. Acad Med. 2022; 97(9): 1413–22 10.1097/ACM0000000000004641 PMID: 35234716. 35234716

[7] Strout TD, Hillen M, Gutheil C et al., Tolerance of uncertainty: A systematic review of health and healthcare-related outcomes. Patient Educ Couns. 2018;101(9):1518–37. 10.1016/j.pec.2018.03.030 PMID: 29655876. 29655876

[8] Hancock J, Mattick K. Tolerance of ambiguity and psychological well-being in medical training: A systematic review. Med Educ. 2020; 54(2): 125–37 [ 10.1111/medu14031 PMID: 31867801. PMC7003828 31867801

[9] Kim K, Lee Y-M. Understanding uncertainty in medicine: concepts and implications in medical education. Korean J Med Educ. 2018;30(3):181–8. 10.3946/kjme.2018.92 PMID: 30180505. PMC6127608 30180505

[10] Geller G, Faden RR, Levine DM. Tolerance for ambiguity among medical students: implications for their selection, training and practice. Soc Sci Med. 1990;31(5):619–24. 10.1016/0277-9536(90)90098-d. PMID: 2218644. 2218644

[11] Nevalainen MK, Mantyranta T, Pitkala KH. Facing uncertainty as a medical student–a qualitative study of their reflective learning diaries and writings on specific themes during the first clinical year. Patient Educ Couns. 2010;78(2):218–23. 10.1016/j.pec.2009.07.011. PMID: 19767167. 19767167

[12] Han PKJ, Schupack D, Daggett S, Holt CT, Strout TD. Temporal changes in tolerance of uncertainty among medical students: insights from an exploratory study. Med Educ Online. 2015;20:28285. 10.3402/meo.v20.28285.PMID: 26356230. PMC4565063 26356230

[13] Stephens GC, Rees CE, Lazarus MD. Exploring the impact of education on preclinical medical students’ tolerance of uncertainty: a qualitative longitudinal study. Adv Health Sci Educ Theory Pract. 2021;26(1):53–77. 10.1007/s10459-020-09971-0.PMID: 32378150. 32378150

[14] Yap A, Johanesen P, Walsh C. Moderators uncertainty tolerance (UT) in healthcare: a systematic review. Adv Health Sci Educ Theory Pract. 2023;28(5):1409–40. 10.1007/s10459-023-10215-0.PMID: 37097482. PMC10700225 37097482

[15] Stephens GC, Lazarus MD, Sarkar M, Karim MN, Wilson AB. Identifying validity evidence for uncertainty tolerance scales: A systematic review. Med Educ. 2023;57(9):844–56 10.1111/medu15014 PMID: 36576391. 36576391

[16] Stephens GC, Sarkar M, Lazarus MD. ‘A whole lot of uncertainty’: A qualitative study exploring clinical medical students’ experiences of uncertainty stimuli. Med Educ 2022; 56(7): 736–46 10.1111/medu14743 PMID: 35130579. PMC9306844 35130579

[17] Stephens GC, Sarkar M, Lazarus MD. Medical Student Experiences of Uncertainty Tolerance Moderators: A Longitudinal Qualitative Study. Front Med (Lausanne). 2022;9:864141. 10.3389/fmed2022.864141.PMID: 35547203. PMC9083353 35547203

[18] DeForge BR, Sobal J. Intolerance of ambiguity in students entering medical school. Soc Sci Med. 1989;28(8):869–74. 10.1016/0277-9536(89)90117-2.PMID: 2705020. 2705020

[19] Nevalainen M, Kuikka L, Sjoberg L, Eriksson J, Pitkala K. Tolerance of uncertainty and fears of making mistakes among fifth-year medical students. Fam Med. 2012;44(4):240–6. PMID: 22481152. 22481152

[20] Evans L, Trotter DRM, Jones BG, et al. Epistemology and uncertainty: a follow-up study with third-year medical students. Fam Med. 2012;44(1):14–21. PMID: 22241336. 22241336

[21] Klugman CM, Peel J, Beckmann-Mendez D. Art Rounds: teaching interprofessional students visual thinking strategies at one school. Acad Med. 2011;86(10):1266–71. 10.1097/ACM.0b013e31822c1427.PMID: 21869658. 21869658

[22] Mann K, Gordon J, MacLeod A. Reflection and reflective practice in health professions education: a systematic review. Adv Health Sci Educ Theory Pract. 2009;14(4):595–621. 10.1007/s10459-007-9090-2.PMID: 18034364. 18034364

[23] Sandars J. The use of reflection in medical education: AMEE Guide No. 44. Med Teach. 2009;31(8): 685–95. 10.1080/01421590903050374. PMID: 19811204. 19811204

[24] Wald HS, Borkan JM, Taylor JS, Anthony D, Reis SP. Fostering and evaluating reflective capacity in medical education: developing the REFLECT rubric for assessing reflective writing. Acad Med. 2012;87(1):41–50. 10.1097/ACM.0b013e31823b55.fa PMID: 22104060. 22104060

[25] Mamede S, Schmidt HG, Penaforte JC. Effects of reflective practice on the accuracy of medical diagnoses. Med Educ. 2008;42(5):468–75. 10.1111/j.1365-2923.2008.03030.x.PMID: 18412886. 18412886

[26] Karnieli-Miller O, Michael K, Gothelf AB, Palombo M, Meitar D. The associations between reflective ability and communication skills among medical students. Patient Educ Couns. 2021; 104(1): 92–8. 10.1016/j.pec.2020.06.028 PMID: 32624329. 32624329

[27] Leung KCY, Peisah CA, Mixed-Methods, Systematic Review of Group Reflective Practice in Medical Students. Healthcare (Basel). 2023; 11(12). 10.3390/healthcare11121798.PMID:37372916. PMC10297932 37372916

[28] Khoshgoftar Z, Barkhordari-Sharifabad M. Medical students’ reflective capacity and its role in their critical thinking disposition. BMC Med Educ. 2023;23(1):198. 10.1186/s12909-023-04163-x.PMID: 36998069. PMC10061695 36998069

[29] Bari A, Imran I, Ullah H, Arshad A, Naeem I, Sadaqat N. Reflection as a Learning Tool in Postgraduate Medical Education. J Coll Physicians Surg Pak. 2021;31(9):1094–8. 10.29271/jcpsp.2021.09.1094. PMID: 34500528. 34500528

[30] Faihs V, Heininger S, McLennan S, Gartmeier M, Berberat PO, Wijnen-Meijer M. Professional Identity and Motivation for Medical School in First-Year Medical Students: A Cross-sectional Study. Med Sci Educ. 2023;33(2):431–41. 10.1007/s40670-023-01754-7.PMID: 37261015. PMC10226964 37261015

[31] Rostami A, Keshmiri F, Askari R, Jambarsang S, Shafiei M. Validation of Groningen Reflection Ability Scale Questionnaire and Evaluation of Reflection Ability Level of Health Care Management Students. JEBHPME. 2019. 10.18502/jebhpme.v3i4.2071.

[32] Dalbert C. Die Ungewißheitstoleranzskala: Skaleneigenschaften und Validierungsbefunde. Leibniz Institut für Psychologie (ZPID); 1999.

[33] Gallo V, Egger M, McCormack V et al., STrengthening the Reporting of OBservational studies in Epidemiology: Molecular Epidemiology STROBE-ME. An extension of the STROBE statement. J Epidemiol Community Health. 2012;66(9):844–54. 10.1136/jech-2011-200318*.*PMID: 22025194. 22025194

[34] Saxena S, Wright WS, Khalil MK. Gender differences in learning and study strategies impact medical students’ preclinical and USMLE step 1 examination performance. BMC Med Educ. 2024;24(1):504. 10.1186/s12909-024-05494-z.PMID: 38714975. PMC11077801 38714975

[35] Sethi I, Mastrogiacomo C, Baldelli P, Wackett A, Abdel-Misih S. Gender-Based Differences in Medical Student Self-Ratings of Clinical Performance. J Surg Res. 2024;302:286–92. 10.1016/j.jss.2024.07.047 PMID: 39116828. 39116828

[36] Madrazo L, Lee CB, McConnell M, Khamisa K. Self-assessment differences between genders in a low-stakes objective structured clinical examination (OSCE). BMC Res Notes. 2018;11(1):393. 10.1186/s13104-018-3494-3.PMID: 29903050. PMC6003209 29903050

[37] Stanek K, Phillips N, Staffa SJ, Saldanha FYL, Rogers-Vizena CR. Gender Differences in Plastic Surgery Trainee Confidence: A Pilot Analysis During Cleft Lip Simulation. Plast Reconstr Surg Glob Open. 2023;11(12):e5428. 10.1097/GOX0000000000005428.PMID:38074498. PMC10703114 38074498

[38] Peugh JL, Enders CK. Missing Data in Educational Research: A Review of Reporting Practices and Suggestions for Improvement. Rev Educ Res. 2004;74(4):525–56. 10.3102/00346543074004525.

[39] Mader EM, Roseamelia C, Morley CP. The temporal decline of idealism in two cohorts of medical students at one institution. BMC Med Educ. 2014;14:58. 10.1186/1472-6920-14-58.PMID: 24655727. PMC3994310 24655727

