# Supplementary material for: Tolerance of uncertainty and reflection ability among medical students in Germany
Source: BMC Med Educ. 2026 Sep 23;26:1517. doi: 10.1186/s12909-026-10455-9 (PMC13602493; doi:10.1186/s12909-026-10455-9)
Supplement: Supplementary file 1 [file 12909_2026_10455_MOESM1_ESM.docx]

# **Supplement**

| German item used in questionnaire | English |
| --- | --- |
| Uncertainty Tolerance Scale | |
| Ich probiere gerne Dinge aus, auch wenn nicht immer etwas dabei herauskommt. | I like to try things out, even if nothing comes out of it. |
| Ich beschäftige mich nur mit Aufgaben, die lösbar sind. | I like to engage in tasks for which there is a solution. |
| Ich mag es, wenn unverhofft Überraschungen auftreten. | I like unexpected surprises. |
| Ich lasse die Dinge gerne auf mich zukommen. | I like to let things happen. |
| Ich habe es gerne, wenn die Arbeit gleichmäßig verläuft. | When I work or study, I like things to be predictable and routine. |
| Ich warte geradezu darauf, dass etwas Aufregendes passiert. | I like change and excitement. |
| Wenn um mich herum alles drunter und drüber geht, fühle ich mich so richtig wohl. | When things around me are all topsy-turvy, I am quite all right. |
| Ich weiß gerne, was auf mich zukommt. | I like to know what to expect. |
| Groningen Reflection Ability Scale | |
| Ich will wissen, warum ich das mache, was ich mache. | I want to know why I do what I do. |
| Die Emotionen, die mein Handeln beeinflussen, sind mir bewusst. | I am aware of the emotions that influence my behavior. |
| Ich mag es nicht, meine eigenen Ansichten zur Diskussion zu stellen. | I do not like to have my standpoints discussed. |
| Ich reagiere abweisend auf Bemerkungen über mein persönliches Handeln. | I do not welcome remarks about my personal functioning. |
| Ich reflektiere meine eigenen Denkweisen. | I take a closer look at my own habits of thinking. |
| Ich kann mein eigenes Verhalten aus Distanz beurteilen. | I am able to view my own behavior from a distance. |
| Ich überprüfe meine Urteile anhand der Urteile anderer Menschen. | I test my own judgments against those of others. |
| Andere sagen manchmal, dass ich mich selbst überschätze. | Sometimes others say that I do overestimate myself. |
| Ich finde es wichtig zu wissen, worauf bestimmte Regeln und Vorschriften beruhen. | I find it important to know what certain rules and guidelines are based on. |
| Ich kann mich in Menschen mit unterschiedlichem kulturellem oder religiösem Hintergrund einfühlen. | I am able to understand people with a different cultural / religious background. |
| Ich übernehme die Verantwortung für meine Aussagen. | I am accountable for what I say. |
| Ich lehne ein anderes Denken ab. | I reject different ways of thinking. |
| Ich kann eine Erfahrung aus verschiedenen Blickwinkeln betrachten. | I can see an experience from different standpoints. |
| Ich kümmere mich um die Aussagen, die ich mache. | I take responsibility for what I say. |
| Ich kann meine eigenen Ansichten in Frage stellen. | I am open to discussion about my opinions. |
| Ich kenne meine Grenzen. | I know my limitations. |
| Manchmal fällt es mir schwer, einen ethischen Standpunkt zu erklären. | I sometimes find myself having difficulty in illustrating an ethical standpoint. |
| Ich bin mir der kulturellen Einflüsse auf meine Ansichten bewusst. | I am aware of the cultural influences on my opinions. |
| Ich will mich selbst verstehen. | I want to understand myself. |
| Ich bin mir darüber bewusst, dass eine Information für Andere emotional geladen sein kann. | I am aware of the possible emotional impact of information on others. |
| Manchmal fällt es mir schwer, alternative Lösungen zu finden. | I sometimes find myself having difficulty in thinking of alternative solutions. |
| Ich kann mich in die Situation eines Anderen hineinversetzen. | I can empathize with someone else’s situation. |
| Die Emotionen, die mein Denken beeinflussen, sind mir bewusst. | I am aware of the emotions that influence my thinking. |

**Supplemental Table 1:** German Items used in the questionnaire and English items as used in the literature.
