# Supplementary material for: Tolerance of uncertainty and reflection ability among medical students in Germany
Source: BMC Med Educ. 2026 Sep 23;26:1517. doi: 10.1186/s12909-026-10455-9 (PMC13602493; doi:10.1186/s12909-026-10455-9)
Supplement: Supplementary file 2 [file 12909_2026_10455_MOESM2_ESM.docx]

| Predictor | B | SE | ß | t | P |
| --- | --- | --- | --- | --- | --- |
| I want to know why I do what I do. **(N=356),** R² 0.012, F (df) 2.150 (2, 353), p (model) 0.118 | | | | | |
| Gender (female) | -0.090 | 0.073 |  | -1.235 | 0.218 |
| Age | 0.017 | 0.010 | 0.085 | 1.604 | 0.110 |
| I am aware of the emotions that influence my behavior. **(N=360)**, R² 0.006, F(df) 1.149 (2, 357), p (model) 0.318 | | | | | |
| Gender (female) | -0.088 | 0.087 |  | -1.018 | 0.309 |
| Age | 0.013 | 0.012 | 0.056 | 1.066 | 0.287 |
| I do not like to have my standpoints discussed. **(N=353)**, R² 0.034, F(df) 6.140 (2, 350), p (model) 0.002 | | | | | |
| Gender (female) | 0.355 | 0.128 |  | 2.779 | 0.006 |
| Age | 0.041 | 0.018 | 0.120 | 2.275 | 0.024 |
| I do not welcome remarks about my personal functioning. **(N=355)**, R² 0.003, F(df) 0.496 (2, 352), p (model) 0.609 | | | | | |
| Gender (female) | 0.103 | 0.104 |  | 0.988 | 0.324 |
| Age | -0.001 | 0.015 | -0.004 | -0.080 | 0.936 |
| I take a closer look at my own habits of thinking. **(N=356)**, R² 0.009, F(df) 1.680 (2, 353), p (model) 0.188 | | | | | |
| Gender (female) | -0.067 | 0.081 |  | -0.827 | 0.409 |
| Age | 0.018 | 0.012 | 0.084 | 1.587 | 0.113 |
| I am able to view my own behavior from a distance. **(N=353)**, R² 0.009, F(df) 1.6212(2, 350), p (model) 0.199 | | | | | |
| Gender (female) | -0.099 | 0.093 |  | -1.060 | 0.290 |
| Age | 0.018 | 0.013 | 0.074 | 1.388 | 0.166 |
| I test my own judgments against those of others. **(N=351)**, R² 0.000, F(df) 0.030 (2, 348), p (model) 0.971 | | | | | |
| Gender (female) | -0.003 | 0.109 |  | -0.028 | 0.978 |
| Age | 0.004 | 0.015 | 0.013 | 0.241 | 0.809 |
| Sometimes others say that I do overestimate myself**. (N=350)**, R² 0.074, F(df) 13.88 (2, 347), p (model) <0.001 | | | | | |
| Gender (female) | -0.570 | 0.108 |  | -5.267 | <0.001 |
| Age | -0.007 | 0.015 | -0.025 | -0.484 | 0.629 |
| I find it important to know what certain rules and guidelines are based on. **(N=353)**, R² 0.002, F(df) 0.430 (2, 350), p (model) 0.651 | | | | | |
| Gender (female) | -0.065 | 0.088 |  | -0.741 | 0.459 |
| Age | 0.006 | 0.013 | 0.028 | 0.515 | 0.607 |
| I am able to understand people with a different cultural / religious background. **(N=349)**, R² 0.002, F(df) 0.391 (2, 346), p (model) 0.676 | | | | | |
| Gender (female) | 0.054 | 0.090 |  | 0.597 | 0.551 |
| Age | -0.008 | 0.013 | -0.033 | -0.614 | 0.540 |
| I am accountable for what I say. **(N=353)**, R² 0.008, F(df) 1.437 (2, 350), p (model) 0.239 | | | | | |
| Gender (female) | -0.126 | 0.075 |  | -1.689 | 0.092 |
| Age | -0.003 | 0.011 | -0.013 | -0.244 | 0.807 |
| I reject different ways of thinking. **(N= 351),** R² 0.020, F(df) 4.513 (2, 348), p (model) 0.012 | | | | | |
| Gender (female) | -0.237 | 0.079 |  | -2.980 | 0.003 |
| Age | 0.002 | 0.011 | 0.012 | 0.218 | 0.827 |
| I can see an experience from different standpoints. **(N=350)**, R² 0.011, F(df) 1.895 (2, 347), p (model) 0.152 | | | | | |
| Gender (female) | 0.025 | 0.076 |  | 0.334 | 0.739 |
| Age | 0.021 | 0.011 | 0.104 | 1.935 | 0.054 |
| I take responsibility for what I say. **(N=348)**, R² 0.010, F(df) 1.717 (2, 345), p (model) 0.181 | | | | | |
| Gender (female) | 0.090 | 0.078 |  | 1.155 | 0.249 |
| Age | 0.017 | 0.011 | 0.081 | 1.507 | 0.133 |
| I am open to discussion about my opinions. **(N=329)**, R² 0.002, F(df) 0.330 (2, 326), p (model) 0.719 | | | | | |
| Gender (female) | 0.052 | 0.075 |  | 0.695 | 0.487 |
| Age | 0.005 | 0.011 | 0.027 | 0.485 | 0.628 |
| I know my limitations. **(N=338)**, R² 0.010, F(df) 1.715 (2, 335), p (model) 0.182 | | | | | |
| Gender (female) | 0.183 | 0.099 |  | 1.848 | 0.065 |
| Age | 3.615x10^-4^ | 0.016 | -0.021 | -0.244 | 0.807 |
| I sometimes find myself having difficulty in illustrating an ethical standpoint. **(N=327)**, R² 0.003, F(df) 0.535 (2, 324), p (model) 0.586 | | | | | |
| Gender (female) | 0.054 | 0.133 |  | 0.408 | 0.684 |
| Age | -0.017 | 0.019 | -0.051 | -0.919 | 0.359 |
| I am aware of the cultural influences on my opinions. **(N=339)**, R² 0.001, F(df) 0.226 (2, 336), p (model) 0.798 | | | | | |
| Gender (female) | 0.059 | 0.096 |  | 0.620 | 0.536 |
| Age | 0.004 | 0.013 | 0.017 | 0.306 | 0.760 |
| I want to understand myself. **(N=333)**, R² 0.016, F(df) 2.665 (2, 330), p (model) 0.071 | | | | | |
| Gender (female) | 0.193 | 0.087 |  | 2.227 | 0.027 |
| Age | -0.005 | 0.012 | -0.024 | -0.438 | 0.662 |
| I am aware of the possible emotional impact of information on others. **(N=332)**, R² 0.015, F(df) 2.532 (2, 329), p (model) 0.081 | | | | | |
| Gender (female) | 0.182 | 0.081 |  | 2.236 | 0.026 |
| Age | -0.001 | 0.011 | -0.005 | -0.092 | 0.927 |
| I sometimes find myself having difficulty in thinking of alternative solutions**. (N=328)**, R² 0.009, F(df) 1.414 (2, 325), p (model) 0.245 | | | | | |
| Gender (female) | 0.150 | 0.112 |  | 1.334 | 0.183 |
| Age | 0.017 | 0.016 | 0.061 | 1.101 | 0.272 |
| I can empathize with someone else’s situation. **(N=326)**, R² 0.017, F(df) 2.732 (2, 323), p (model) 0.067 | | | | | |
| Gender (female) | 0.162 | 0.083 |  | 1.950 | 0.052 |
| Age | 0.017 | 0.012 | 0.078 | 1.412 | 0.159 |
| I am aware of the emotions that influence my thinking. **(N=330)**, R² 0.002, F(df) 0.270 (2, 327), p (model) 0.763 | | | | | |
| Gender (female) | 0.021 | 0.092 |  | 0.228 | 0.820 |
| Age | 0.009 | 0.013 | 0.040 | 0.712 | 0.477 |

Supplemental Table 2: Relationship between age / gender and Reflection ability, results of linear regression analysis. B: unstandardized coefficient, ß: standardized coefficient, R²: coefficient of determination, indicating the proportion of variance in the dependent variable explained by the regression model, F(df): F-statistic with degrees of freedom. 377 questionnaires were included, the respective number of analyzed questionnaires per item is given as **N**.
